# A Comparison of the Ability Emotional Intelligence of Head Teachers With School Teachers in Other Positions

**DOI:** 10.3389/fpsyg.2019.00841

**Published:** 2019-04-17

**Authors:** María José Gutiérrez-Cobo, Rosario Cabello, Juan Rodríguez-Corrales, Alberto Megías-Robles, Raquel Gómez-Leal, Pablo Fernández-Berrocal

**Affiliations:** ^1^Department of Developmental and Educational Psychology, University of Granada, Granada, Spain; ^2^Department of Basic Psychology, Faculty of Psychology, University of Málaga, Málaga, Spain

**Keywords:** head teacher, teacher, emotional intelligence, MSCEIT, leadership

## Abstract

Head teachers are exposed to a highly emotional and stressful job, and they need a sufficient combination of professional competencies in order to deal with daily challenges in schools. Recent studies have shown the importance of developing emotional competencies such as emotional intelligence (EI) in teachers in order to improve their professional development and to ensure the adequate functioning of the school. However, rather less is known about the ability EI of head teachers. The aim of the present study was to evaluate the ability EI of public school head teachers and compare this ability with those working in other positions within the school. For these purposes, 393 participants (35 head teachers, 39 middle leaders, 236 tutors, and 86 teachers) aged between 24 and 62 years (*M* = 40.26; *SD* = 9.27) completed the mayer-salovey-caruso emotional intelligence test (MSCEIT). The results revealed a significantly higher total EI for head teachers than teachers, along with higher scores in the understanding branch of the MSCEIT for the head teachers compared with workers in other positions. In addition, on this EI branch, tutors also achieved higher scores than the teachers. We also evaluated the alternative hypothesis that years of teaching experience could explain the relationship between work position and the EI scores, and found no evidence in support of this possibility. Limitations and future lines of research are discussed.

## Introduction

The school setting is composed of an interactive network of actors that influence each other. Among them, particularly noteworthy is the role played by teachers and head teachers, given their influence on students and the adequate functioning of the school (Frenzel et al., 2009; Jiang et al., 2016; Sánchez-Rosas et al., 2016).

Teaching is a highly emotional and stressful job (Chang, 2009; Brackett et al., 2010; Keller et al., 2014). In addition to this, head teachers are required to deal with task and time management, inefficient staff, discipline and support of teachers, loneliness, and new educational policies (Hobson, 2003; Starr, 2011; López et al., 2012; Page, 2016). Therefore, the role of teachers — and particularly that of the head teacher — requires physical, intellectual, and emotional energy (Harris, 2007).

Stress can have an impact on the way in which the teacher is able to function in his/her role. In particular, stress is related to negative outcomes such as lower job satisfaction, mental health problems, identity and personal accomplishment or effectiveness at work (Beehr, 2014; Formica et al., 2017). Therefore, it is of importance to search for variables that could play a protective role, which has been the focus of a number of previous studies. The literature has found that variables such as personal and collective efficacy at work, personality characteristics such as extraversion, and proper coping strategies are all linked to a reduction in stress (Kokkinos, 2016; Zurlo et al., 2016; Ramaci et al., 2017).

Given these previous findings there is no doubt about the important role played by emotions in the organization and adequate development of the school (Crawford, 2007a,b; Britan et al., 2013; Rodríguez-Corrales et al., 2017; Pekrun et al., 2018) and there is clearly a need to implement programs to improve these competencies (Castillo-Gualda et al., 2019).

The role of head teachers, as leaders in the school organization, is vital for the functioning of the institution. In general, leaders must have the capacity to generate results by inspiring and motivating their workers. For these tasks, leaders require high levels of emotional competency in order to achieve the best possible results and encourage development of the company, or in our case, the school (Forgas and George, 2001). All these characteristics form the basis of transformational leadership, which has been shown to be the best predictor of leader effectiveness (Ayro, 2014). In this style of leadership, the emotional intelligence (EI) concept has great significance, since there is a strong correlation between EI and the effectiveness of transformational leaders (Barling et al., 2000; Mandell and Pherwani, 2003; Leban and Zulauf, 2004). In particular, higher scores on the management branch of EI are related to higher scores on several factors of transformational leadership (Palmer et al., 2001; Ayro, 2014).

Emotional intelligence is conceptualized by Mayer and Salovey (1997) as a hierarchical model of four branches of increasing complexity: perceiving, facilitating, understanding, and managing emotions. Mayer and Salovey (1997, pp.10) define it as “…the ability to perceive accurately, appraise, and express emotion; the ability to access and/or generate feelings when they facilitate thought; the ability to understand emotion and emotional knowledge; and the ability to regulate emotions to promote emotional and intellectual growth.”

Emotional intelligence has been theoretically conceptualized according to the following three main approaches (Joseph and Newman, 2010): performance-based ability model, self-report ability model, and self-report mixed model. The Performance-based ability model understands EI to be a form of intelligence based on a set of emotional aptitudes measured through objective instruments where individuals have to solve emotional problems. The Self-report ability model also describes EI as a form of intelligence, but it uses subjective measures where participants indicate their perception of their own EI. Finally, self-report mixed models understand EI as a broader construct composed of among others motivations, interpersonal and intrapersonal abilities, empathy and emotional aptitudes, and again employs self-reports to measure the construct.

Although the three main approaches to EI are frequently employed in research, they do not appear to be strongly inter-correlated (Goldenberg et al., 2006; Webb et al., 2013; Gutiérrez-Cobo et al., 2017). This means that whilst an individual may have the perception of being high in EI (as measured by self-report), they could still have a low score on the performance test, and vice versa. In addition, in comparison with self-report models, the performance-based ability model relies more heavily on empirical support (Mayer et al., 2016). We will therefore focus on the performance-based ability model in the present study. One of the most important instruments for evaluating EI through this model is the mayer-salovey-caruso emotional intelligence test (MSCEIT, Mayer et al., 2002). The MSCEIT evaluates EI following the Mayer and Salovey (1997) EI conceptualization and, therefore, evaluates each of its 4 branches along with a global EI index. However, the MSCEIT is not without criticism (Matthews et al., 2004; Maul, 2012; Fiori et al., 2014). For instance, Fiori et al. (2014) showed that this test could fail to discriminate between moderate and higher EI participants, being only suitable for those with deficiencies in EI.

In the general population, EI — as measured through the three main approaches — has been shown to be beneficial for well-being, mental and physical health, and job performance, among other aspects (Joseph and Newman, 2010; Martins et al., 2010; Zeidner et al., 2012; Cabello and Fernández-Berrocal, 2015). In addition, gender differences are usually found in EI, with females scoring higher than males (Cabello et al., 2016). In teachers, EI has also been shown to be a protective factor for well-being, job satisfaction, and engagement (Yin et al., 2013; Fernández-Berrocal et al., 2017; Mérida-López and Extremera, 2017). However, rather less is known about the relevance of the EI construct in head teachers. To our knowledge, only one study has examined EI scores in this population (Benson et al., 2014). These authors analyzed EI through a self-report mixed model instrument and found differences between senior and middle school leaders (a directional school position in which the leader is in charge of organizing only a specific school cycle). In particular, it was found that senior leaders achieved a higher EI score than the middle leaders.

It is important to consider Spanish legislation in order to understand how head teachers are selected in this country. The process begins with the opening of a call, where those teachers interested in the head teacher position must complete a specific application together with a direction project and a list of merits (BOJA N° 222, 2017). The best candidates are then chosen for the positions. In this context, it would be interesting to elucidate if those candidates chosen have higher EI or if this is acquired during the course of their role as head teacher.

The aim of the present study was to analyze the ability EI profile — as measured through the MSCEIT — of public school Spanish head teachers in comparison with teachers with no head positions and middle leaders. We hypothesized that head teachers will present higher EI compared with teachers, since head teachers are required to manage a wider and more complex number of tasks. Given that head teachers play the role of leaders in their educational centers, it is expected that among their characteristics they will show high EI, which will help them to exercise their role in the most efficient way possible. However, since a previous study found that teaching experience influences the scores of teachers on EI (Benson et al., 2014), higher scores on EI for head teachers could be due to having more extensive teaching experience, rather than their work position. We will therefore explore the alternative hypothesis that years of teaching experience could explain the relationship between work position and EI scores. Finally, as a secondary aim of our study, gender differences will be analyzed.

Our investigation addresses some key aspects that have not been considered in other studies. First, a considerable number of previous studies have employed a sample of future teachers that are not yet working in the profession (e.g., Gutiérrez-Moret et al., 2016). In contrast, we recruited an extensive sample of individuals working at the school (from head teacher to teachers without leading positions). Second, many studies have primarily employed subjective self-report measures (e.g., Fernández-Berrocal et al., 2017; Latif et al., 2017; Mérida-López et al., 2017), whilst we use the most characteristic performance test of EI: the MSCEIT. Third, with respect to head teacher samples, few studies evaluate this position in the EI field and the majority are qualitative or theoretical studies that use samples of approximately five participants (Crawford, 2007a,b). Our study, on the other hand, employs a larger sample as well as a quantitative design. Finally, to our knowledge, the present study is the first to evaluate the EI of Spanish head teachers.

## Materials and Methods

### Participants and Procedure

The sample was composed of 393 participants (74.81% women): 35 head teachers (71.4% women), 39 middle leaders (61.5% women), 236 tutors (77.1% women), and 83 teachers with no tutoring responsibilities (75.9% women). Participants were recruited from employees of 35 Spanish public schools that attended SEL-sponsored courses developed by the Regional Government of Andalusia between the years 2014 and 2016. All of the attendees participated in the study (a rate of 100%). The age of the participants ranged from 24 to 62 years with an average of 40.26 (*SD* = 9.27). The average number of years of teaching experience was 11.99 (*SD* = 10.01). All participants completed the test of the study for 45 min in the same quiet room.

The study was carried out in accordance with the Declaration of Helsinki and ethical guidelines of the American Psychological Association, and all participants provided written informed consent. The Research Ethics Committee of the University of Málaga approved the study protocol as part of the projects SEJ-07325.

### Instruments

Mayer-salovey-caruso emotional intelligence test (Mayer et al., 2002; Extremera and Fernández-Berrocal, 2009). We employed the Spanish version of this performance-based ability test that shows adequate psychometric properties similar to the English language version (Cronbach’s α = 0.95; Sánchez-Garcia et al., 2016). This instrument is composed of 141 items. It offers a separate score for each of the four EI branches following the Mayer and Salovey (1997) approach (perceiving, facilitating, understanding, and managing emotions) and a total score. The Perceiving emotion score reflects the ability to perceive emotions in one’s self and others as well as in other stimuli (e.g., objects or art). Facilitating emotion scores reveal the capacity to generate, use, and feel emotions that are necessary to communicate feelings, or employ them in other cognitive processes. The third score is the Understanding emotion branch, which refers to the ability to understand emotional information, how emotions combine and progress through relationship transitions, and to appreciate such emotional meanings. Finally, the Managing emotion branch reflects the ability to modulate emotions in one’s self and others to promote personal understanding and growth. Each of the four branches is assessed by two tasks. An example of an item, in this case related to the managing branch, would be to describe an individual emotional state after which participants have to choose, on a scale from 1 (very ineffective) to 5 (very effective), how different activities would preserve this mood. In the present study, internal consistency ranged from 0.70 to 0.88 (perceiving = 0.85; facilitating = 0.70; understanding = 0.72; managing = 0.72; and MSCEIT total = 0.88). This consistency was measured using Cronbach’s alpha in the case of MSCEIT total score, and the split-halves consistency test in the case of the branches.

### Data Analyses

All statistical analyses were carried out using the SPSS package (version 20.0; IBM, United States). Preliminary analyses were conducted to compute descriptive statistics (mean and standard deviation) of the study variables. A *t*-tests was then carried out to analyze gender differences in EI scores (MSCEIT global, perceiving, facilitating, understanding, and managing). A series of one-way ANOVAs were then conducted to analyze differences in EI scores between work position (head teacher, middle leader, tutor, and teacher). In this latter analysis, we did not evaluate the interaction gender x working position given the higher number of women than men in our sample (e.g., for head teachers: 10 men as opposed to 25 women). Finally, in order to analyze the alternative hypothesis that years of teaching experience could explain the relationship between work position and EI scores, correlational analyses were carried out between years of teaching experience and the MSCEIT global score and branches. In addition, a one-way ANCOVA analysis was conducted with work position as the independent variable, with the understanding branch of the MSCEIT as the dependent variable, and years of teaching experience as a covariate.

## Results

**FIGURE 1:**
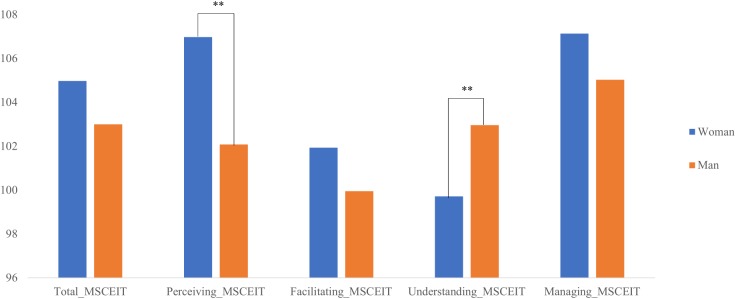
Differences in emotional intelligence according to gender. ^∗∗^*p* < 0.01.

Descriptive statistics for the study variable (MSCEIT total, perceiving, facilitating, understanding and managing) in work position and gender as well as for years of teaching experience in work position are shown in Table 1. In order to understand these descriptive statistics, it is important to take into account that scores under 90 in EI are indicative of a low EI level; scores between 90 and 110 indicate a competent level; scores between 110 and 130 indicate a very competent level and, finally, scores higher than 130 are associated with EI experts. It can be observed that each participant, independently of work position, showed a competent EI level (scores between 90 and 110). We also analyzed gender differences in EI. We conducted *t*-test comparison for each EI branch and for the total score (Figure 1). These analyses revealed differences in the perceiving and the understanding branches. In particular, for the perceiving branch, women showed higher scores than men, *t*(391) = 3.41, *p* < 0.01, *d*_z_ = 0.04; whilst for the understanding branch, men achieved higher scores than women, *t*(391) = 2.61, *p* < 0.01, *d*_z_ = 0.01, although these gender differences are of a small effect size. No other significant results were found (facilitating, *p* = 0.12; managing, *p* = 0.12, and total, *p* = 0.07).

**Table 1:** Mean and standard deviation of the study variables.

	Teaching experience	MSCEIT Total	MSCEIT Perceiving	MSCEIT Facilitating	MSCEIT Understanding	MSCEIT Managing
Headmasters	18.60 (10.50)	106.42 (8.46)	105.65 (13.44)	102.83 (8.01)	105.35 (8.49)	106.82 (11.53)
Middle leaders	17.31 (10.23)	103.33 (8.14)	105.51 (12.08)	100.31 (9.64)	100.36 (13.76)	103.91 (13.70)
Tutors	10.82 (9.76)	105.14 (9.05)	106.04 (12.64)	102.28 (10.44)	101.01 (10.18)	107.22 (11.37)
Teachers	10.01 (8.47)	102.32 (10.14)	105.07 (12.28)	98.00 (13.05)	97.25 (97.25)	106.03 (11.01)
Female		104.98 (9.33)	106.98 (11.88)	101.94 (10.98)	99.72 (11.25)	107.14 (11.49)
Male		102.10 (8.76)	102.08 (13.76)	99.95 (10.33)	102.97 (8.87)	105.03 (11.68)


**FIGURE 2:**
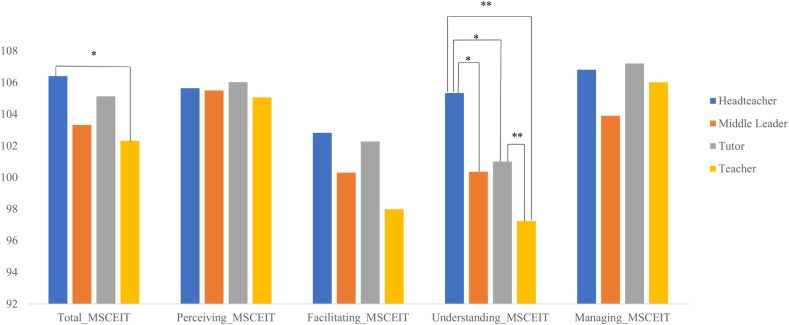
Differences in emotional intelligence according to work position. ^∗^*p* < 0.05; ^∗∗^*p* < 0.01.

Of more interest for our current purposes, we examined differences between the MSCEIT total score according to work position (head teacher, middle leader, tutor, and teacher) by conducting a one-way ANOVA. This revealed a main effect of work position *F*(3,389) = 2.67, *p* < 0.05, *η^2^* = 0.02. *Post hoc* comparisons showed that there was a significant difference in scores between head teachers and teachers with the latter showing lower scores (*p* < 0.05) (Figure 2). Similarly, we conducted a one-way ANOVA for each EI branch according to work position. We found a main effect of work position only for the understanding branch, *F*(3,389) = 5.20, *p* < 0.01, *η^2^* = 0.04. Again, these effects are of a small size. For the other branches, no significant differences were found (perceiving, *p* = 0.95; facilitating, *p* = 0.08, and managing, *p* = 0.39). *Post hoc* analyses for the understanding branch revealed that differences were found between head teachers and middle leaders (*p* < 0.05), tutors (*p* < 0.05) and teachers (*p* < 0.01), with head teachers achieving higher scores. Differences were also found between teachers and tutors (*p* < 0.01), with tutors showing higher scores (Figure 2).

In order to explore the alternative hypothesis that years of teaching experience could explain the relationship found between work position and EI scores, we conducted correlational analyses between years of teaching experience and the MSCEIT global score and branches. This revealed a significant relationship between years of teaching experience and scores on the understanding branch (*r* = 0.10; *p* = 0.045). Thus, a greater number of teaching years correlates with higher scores on the understanding branch of the MSCEIT. No other significant results were found (perceiving, *r* = -0.039, *p* = 0.446; facilitating, *r* = 0.039, *p* = 0.446; managing, *r* = 0.023 *p* = 0.646, and total, *r* = 0.037, *p* = 0.459).

Given that the understanding branch appears to significantly correlate with years of teaching experience, we conducted a one-way ANCOVA with the understanding branch as the dependent variable for work position, and years of teaching experience as a covariate. This analysis revealed a main effect of work position *F*(3,388) = 4.48, *p* < 0.01, *η^2^* = 0.03. No significant results were found for years of teaching experience (*F* = 1.92; *p* = 0.17).

## Discussion

In the present study, we compared the EI profile — as measured through the MSCEIT performance-based test — of Spanish public school head teachers with those of teachers with no leadership positions and those of middle school leaders. We evaluated the EI total score as well as the score for each of the four EI branches: perceiving, facilitating, understanding, and managing branches.

As we hypothesized, head teachers showed higher levels of total EI than teachers, although no significant differences were found between the other school positions. With respect to the EI branches, we found significant differences in the understanding branch of the MSCEIT. In this case, differences were found between head teachers and the rest of the participants: middle leaders, tutors and, again, teachers. These results revealed higher scores for head teachers in comparison with the other positions, findings that are consistent with a previous study using a self-report instrument (Benson et al., 2014). Further, the greatest differences were found between head teachers and the workers with no leadership role and fewer responsibilities, that is, for teachers. In addition, tutors also showed higher scores than teachers on the understanding branch. It is noteworthy that no differences were found between head teachers and teachers in terms of scores on the management branch in spite of this branch being the one that is most predictable for transformational leadership (Palmer et al., 2001; Ayro, 2014).

Previous differences found between head teachers and teachers in the understanding branch could be due to the years of teaching experience, according to the results of Benson et al. (2014). Nonetheless, this variable showed no significant results when introduced as a covariate between the understanding branch and the work position variable. We were thus unable to confirm the alternative hypothesis and the differences in the understanding branch appear to be due to the work position.

Emotional intelligence has already shown to be a protective factor for teachers (Yin et al., 2013; Fernández-Berrocal et al., 2017; Mérida-López and Extremera, 2017). Therefore, given the demanding and emotional nature of the head teacher role (Pekrun et al., 2018), our results, although with a small effect size, are encouraging for this worker’s profile. In addition, considering the Spanish legislation for applying for a head teacher position (BOJA N° 222, 2017), it appears that, in an indirect way, higher EI individuals are selected for the head teacher position. However, an important question arises here: do individuals who apply for the head teacher position and are finally selected have higher EI or do they acquire this ability as a result of dealing with various situations during the course of their head teacher career? Future research should aim to analyze this question using prospective studies.

In spite of head teachers showing more favorable EI compared with teachers, their EI scores, both globally and for the four branches, although indicative of a competent ability, do not reach extremely competent levels. It is possible that this competent level of EI is sufficient to manage daily problems and challenges in schools, but not to lead and generate significant changes in educational organizations. Given the relevance of EI for transformational leadership (Barling et al., 2000; Mandell and Pherwani, 2003; Leban and Zulauf, 2004), participation in EI training programs might be useful in order to achieve higher scores on EI, particularly in terms of the ability to regulate emotions.

Regarding the secondary aim of the present study, although gender differences in EI scores are usually found in the general population with women scoring higher on EI when using MSCEIT (Cabello et al., 2016), the present study only found EI differences in two branches, and not always in the expected direction (although with a small effect size). In particular, even though women scored higher on the perceiving branch as anticipated, they scored lower on the understanding branch. We speculate that the higher scores for men could be due to their vocational interests rather than being representative of the general male population. Nonetheless, this possibility should also be addressed in future work. Another explanation of this result may be due to the imbalance of gender in our sample with almost 75% of our participants being females.

The present study has practical implications at the research level. Firstly, we have recruited active teachers in spite of the common use of samples composed of future teachers (Gutiérrez-Moret et al., 2016). Secondly, the measurement of EI has been conducted through an ability test in order to overcome the limitations of self-report measures (Fernández-Berrocal et al., 2017; Latif et al., 2017; Mérida-López et al., 2017). Thirdly, we offer to the literature a quantitative approach that is scarce in the study of head teachers and EI. Finally, we examined a sufficient sample of head teachers, which is not commonly found in the literature. All of these advantages open up a possible further line of investigation. In particular, as opposed to the case of teachers in which there is evidence of the protective role played by EI (Yin et al., 2013; Fernández-Berrocal et al., 2017; Mérida-López and Extremera, 2017), no such evidence exists for head teachers. It would therefore be of interest to evaluate the role of EI in the well-being and job satisfaction of head teachers, among other variables. For instance, it would be worthwhile to examine the effects of head teacher EI levels on the school environment as well as on teacher and student attainment and well-being. Moreover, future studies should analyze the effect of EI training not only on head teachers but also on other school workers in terms of other positive variables in order to experimentally explore the impact of EI in this environment.

In addition to the research implications of our work, our study has applied clinical relevance. For instance, it would be interesting to implement EI training programs aimed at all school workers, and particularly head teachers, given the higher demands of their role (Hobson, 2003; Starr, 2011; López et al., 2012; Page, 2016). Moreover, universities could include EI training as part of the education program of future teachers, and this could also be included as part of the selection process used by specific schools to choose the best candidates to fill their teaching positions.

The present study is not without limitations. Firstly, although we recruited a large sample, our results are not generalizable to the population. Secondly, our study only includes public school workers, so it would be interesting to analyze private school samples. In addition, although MSCEIT is the most widely used ability EI instrument, it is not without limitations (Matthews et al., 2004; Maul, 2012; Fiori et al., 2014). Thus, any conclusions drawn on the basis of these results should be treated with caution. Finally, gender is not balanced in our sample, making difficult the comparison between males and females. However, this imbalance reflects the real distribution of workers by gender in Spanish schools.

## Conclusion

In conclusion, the present study has revealed the superior EI of head teachers in comparison with teachers without leadership positions in terms of both the total score and the understanding branch of the MSCEIT. To our knowledge this is the first study to evaluate EI in an extensive sample of Spanish head teachers in comparison with that of workers in other positions within the school. In addition, our work represents a departure from the usual subjective self-report measures and instead employs a well-validated performance test of EI, providing quantitative data.

## Ethics Statement

The study was carried out in accordance with the Declaration of Helsinki and ethical guidelines of the American Psychological Association, and all participants (parents and teachers) provided written informed consent. The Research Ethics Committee of the University of Málaga approved the study protocol as part of the projects SEJ-07325.

## Author Contributions

All authors contributed to the conception and design of the study and approved the submitted version and agreed to be accountable for all aspects of the work. MG-C and JR-C contributed to the acquisition of the data. AM-R organized the database. RC, RG-L, and PF-B performed the statistical analysis. MG-C wrote the first draft of the manuscript. RC, JR-C, AM-R, RG-L, and PF-B revised it critically for important intellectual content.

## Conflict of Interest Statement

The authors declare that the research was conducted in the absence of any commercial or financial relationships that could be construed as a potential conflict of interest.
